# Comprehensive two‐dimensional gas chromatography— A discussion on recent innovations

**DOI:** 10.1002/jssc.202300304

**Published:** 2023-08-31

**Authors:** Nino B. L. Milani, Eric van Gilst, Bob W. J. Pirok, Peter J. Schoenmakers

**Affiliations:** ^1^ Van't Hoff Institute for Molecular Science (HIMS) University of Amsterdam Amsterdam the Netherlands

**Keywords:** Complex samples, Comprehensive two‐dimensional gas chromatography, Data analysis, Gas chromatography Mass spectrometry, Modulation

## Abstract

Although comprehensive 2‐D GC is an established and often applied analytical method, the field is still highly dynamic thanks to a remarkable number of innovations. In this review, we discuss a number of recent developments in comprehensive 2‐D GC technology. A variety of modulation methods are still being actively investigated and many exciting improvements are discussed in this review. We also review interesting developments in detection methods, retention modeling, and data analysis.

**Article Related Abbreviations**
μECD: micro electron‐capture detector
^1^D: first‐dimension
1DGC: conventional one‐dimensional gas chromatography
^2^D: second‐dimension
AED: atomic‐emission detection
CI: chemical ionization
DPGM: dynamic pressure gradient modulation
FF: forward‐flush
FI: field‐ionization
FID: flame‐ionization detector
HR: high‐resolution
PI: photo ionization
Q‐ToF: hybrid quadrupole—time‐of‐flight
RFF: reverse fill/flush
SCD: sulphur‐chemiluminescence detector
SNAT: Splitter‐based Non‐cryogenic Artificial Trapping
VUV: vacuum UV

## 1 INTRODUCTION

Unquestionably, comprehensive GC × GC has become a very important technique for characterizing complex samples of volatile analytes. GC × GC constitutes a giant leap in separation power as compared to conventional 1‐D GC, offering peak capacities of tens of thousands instead of a mere thousand. GC × GC also offers structured chromatograms, in which classes of analyte compounds are grouped and (homologous) series and types of isomers can be recognized. This results in highly detailed, interpretable fingerprints of mineral‐oil products. These types of samples spurred the development of 1DGC in the 1950s and then that of GC × GC in the 1990s. For many other types of complex samples, the online combination of 1DGC with MS, that is, GC‐MS, has become the benchmark technique. For the (tentative) identification of specific analytes and for the quantitative analysis of limited numbers of target analytes, the position of GC‐MS is indisputable. However, GC × GC offers undeniable advantages, especially for fingerprinting. GC × GC can be combined online with MS (GC × GC‐MS), but also with near‐universal (eg, flame‐ionization detector, FID) or element‐specific (eg, sulphur‐chemiluminescence detector, SCD) detectors. It offers the separation of numerous isomers and other analytes with equal molecular weights (“isobaric” compounds). Most importantly, GC × GC yields reproducible and readily interpretable fingerprints. As a result, GC × GC has become an established technique in many laboratories. However, there are still many recent and ongoing investigations aimed at improving the GC × GC technique and exploring new applications, indicating that GC × GC, while established, is not yet a mature analytical technique. Especially the scientific and technical advances are the subject of this review.

We speak of a comprehensive 2‐D chromatographic method [1, 2] (i) if either every bit of the sample is subjected to two different separations, or the 2‐D chromatogram obtained is otherwise representative of the entire sample (after eventual pretreatment) and (ii) if the separation (resolution) obtained in the first dimension is essentially maintained. The first requirement can be met in different ways in GC × GC. In many cases, no sample goes to waste (everything passes through the detector). In other approaches to GC × GC, this is not the case, but the 2‐D chromatogram is representative for the entire sample, because there is a constant split of the first‐dimension (^1^D) effluent between the second‐dimension (^2^D) column and waste (or a detector), or ^2^D separations are performed very frequently on small portions of the ^1^D effluent, so as to yield enough data points to describe the ^1^D peaks accurately.

In all cases, comprehensive operation poses demands on the number of ^2^D chromatograms recorded over the span of each ^1^D peak. Bruckner et al. [3] phrased this as “the first column generates chromatograms faster than the second column generates peaks.” To avoid losing ^1^D resolution, it is generally assumed that at least four cuts should be taken per ^1^D peak. Unless the ^1^D gas flow is periodically halted (“stop‐flow” operation), the time between two ^2^D injections, the modulation time or modulation period (*t*_mod_), should not be larger than the standard deviation of the ^1^D peak (ie, *t*_mod_ ≤ ^1^ *σ_t_*). Efficient operation of open‐tubular (capillary) columns requires low retention factors during migration of the analyte [4, 5]. This is generally achieved by using temperature programming. Certainly, the retention factor at the moment of elution will be below unity. This leads to the following criterion:


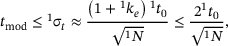

where 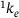 is the retention factor at the moment of elution from the ^1^D column and 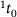 and 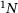 are the dead time and the plate number of the ^1^D column, respectively. High‐resolution (HR) GC columns typically exhibit hold‐up times of a few minutes and plate counts up to 100,000. Such numbers suggest that modulation times of the order of 1 s should be aimed for in GC × GC. Blumberg [6] has deduced that the number of cuts per 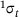, which he defines as the sampling density, may actually be lower (resulting in two or three instead of four cuts per peak). The number of cuts per peak (or per 4⋅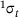 bandwidth) is an intuitively easy concept, which some authors call the modulation ratio (see [7]). Another reason why ^2^D chromatograms may be slightly longer than the 1 s estimate found above is that ^1^D columns and conditions in GC × GC are often selected to yield a slightly lower performance than stand‐alone 1D GC columns. Finally, there is a desire to avoid “wrap‐around,” which occurs if peaks are retained longer in the ^2^D column than the modulation time, causing them to appear in the next cycle, often as broader peaks. As a result, modulation times in the range of 1 to 10 s are typically encountered in GC × GC [8].

The best way to achieve very fast GC analysis is to reduce the column diameter ($d_{c}$). This has a very strong effect, because columns can be shorter (proportional to $d_{c}$) and linear velocities can be higher (proportional to $d_{c}$ *^−1^*). However, when combining columns of different internal diameters (id), the optimal mass flow of carrier gas in these columns is different. This implies that if the same stream of carrier gas is flowing through both columns, a compromise must be struck. Usually, this means that a (relatively broad) ^1^D column is operated around its optimal flow velocity, while a narrower ^2^D column is operated well above the optimum. In some earlier work, a flow splitter was installed between the two columns ([3]), but this is not a broadly accepted approach. A jump down in column diameter by a factor of 2.5 or less (eg, 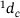 = 250 μm and 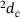 = 100 μm, or 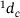 = 320 μm and 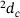 = 150 μm), while maintaining a constant mass flow rate, has generally been found to yield good results.

The most important difference between 1DGC and GC × GC technologies is the need for the effective collection of fractions from the ^1^D effluent and injecting these on the ^2^D column. This process is known as *modulation* and the associated hardware is known as a *modulator*. The main function of the modulator is to turn a peak eluting from the ^1^D column into a series of sharp injection pulses on the ^2^D column. The functions of the modulator can be summarized as follows:

- to sample adjacent, narrow fractions from the ^1^D effluent;
- if possible, to focus these fractions into very narrow pulses;
- to effectively inject these narrow pulses into the ^2^D column.

Very narrow injection pulses are required to avoid losing ^2^D resolution. They also help increase the injection concentration of the analyte in the ^2^D system, thus, enhancing analytical sensitivity and reducing detection limits. Efficient release of collected fractions is also essential to avoid additional band broadening in the ^2^D system. The advent of contemporary GC × GC started with the brilliant inventions of John Phillips in the 1990s. In 1991, he described the first modulator for GC × GC that met the above requirements [9]. This thermal modulator was imperfect. It did not succeed in “trapping” (ie, collecting and focusing) highly volatile analytes (eg, gasoline‐range hydrocarbons) and, most of all, it was not sufficiently robust to routinely perform hundreds of modulations in GC × GC analyses. Much of the recent research on the technology of GC × GC still involves further development of modulators.

Other aspects of GC × GC that are still in the development include detection methods. The most stringent requirements for GC × GC detectors is a very short time constant. For example, MS instruments require very high acquisition rates if they are to be fully compatible with GC × GC. The long strings of very fast ^2^D chromatograms produced in each GC × GC analysis also put severe demands on data handling and data treatment techniques. These strings of chromatograms must be converted to color plots. One important complication is that a truly comprehensive operation implies that a single analyte is spread across a number of ^2^D peaks. In the 2‐D chromatogram, the signals belonging to a single analyte must be merged correctly to ensure correct quantitative analysis [10, 11].

A significant obstacle to the further proliferation of GC × GC is the need for expert knowledge to develop and optimize methods. This has led to significant research efforts devoted to retention modeling and prediction and optimization of GC × GC separations using contemporary software and artificial‐intelligence tools.

In this review, we discuss recent developments in GC × GC technology. Apart from some older articles that help us lay a foundation, nearly three‐quarters of the references (73%) in this review are from the last ten years, while nearly half are from the last five (45%). Much of this has focused on modulation. Some studies into detection methods for GC × GC are also discussed. We also review developments in data handling and computer‐aided method development strategies. We do not review recent applications of GC × GC. The reader is referred to dedicated reviews, focused on food [12, 13, 14], drug discovery [15], environmental [16], petrochemistry [17, 18], and health [19, 20].

## 2 MODULATION

**FIGURE 1:**
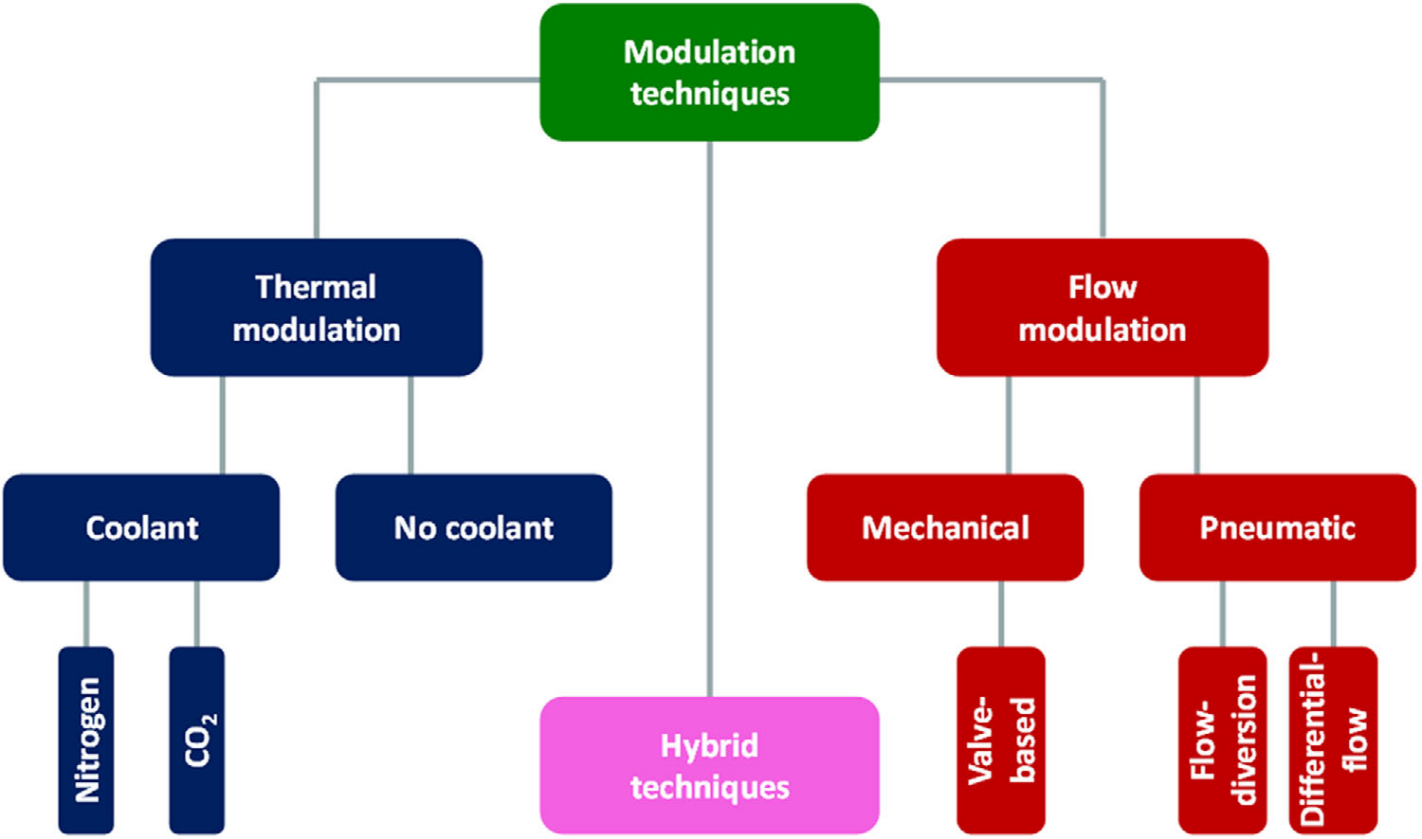
Overview of contemporary modulation techniques for GC × GC.

Without doubt, the modulator is the most‐important hardware component of a GC × GC system. Based on the number of recent explorations of new or improved modulators, it may also be the least‐mature component. Bahaghighat et al. [8] list a number of criteria by which the performance of modulators may be judged. They define (i) duty cycle, (ii) modulation period, (iii) injection pulse width, and (iv) the resulting peak capacity of the ^2^D separation. The duty cycle is defined as the fraction of the ^1^D effluent that is transferred to the ^2^D column. A fifth criterion that may be added is the enhancement factor, that is, (v) the ratio of the analyte concentration after and before modulation. This latter criterion is not independent of the duty cycle (i) and the pulse width (iii), but it is relevant, and other criteria (eg, iii and iv) are also mutually dependent. Figure 1 provides a classification of modulation techniques.

Thermal modulation is the oldest and most common modulation method. It is used in the vast majority of current application papers. However, flow modulators are a hot topic in current GC × GC research (see below). Hybrid modulators that combine thermal and flow effects are used occasionally. The word cryogenic is used rather loosely by chromatographers. In physics, the term is reserved for processes taking place at temperatures below 120 K (−153°C). Some chromatographers use the word *cryogenic* in the original Greek meaning (“frost‐producing”), but this interpretation is not rooted in science. Expanding CO_2_ results in snow with a temperature of −78.5°C, which means it should not be referred to as cryogenic according to the physics definition. Liquid nitrogen has a temperature of −196°C, and it may be used in genuinely cryogenic modulators.

Thermal modulators are based on trapping analytes at a cold spot and releasing them once the spot is heated, either actively (by local heating) or passively (by removing the source of cooling). From the very beginning of GC × GC [9], researchers have realized that two‐stage (or dual‐stage) modulation is the easiest way to fully achieve the objectives of modulation outlined in the introduction. In one‐stage (or single‐stage) modulation, some of the analytes may pass through the modulator without being focused, resulting in poorly shaped peaks or “breakthrough” [7]. Although optimized single‐stage modulators have been used without significant breakthrough [21, 22], the concept of dual‐stage modulation is used in the vast majority of thermal modulators. The earliest thermal modulators used resistive heating [9] or a slotted heater (“sweeper” [9]), and no cooling below the temperature just outside or in the GC oven, respectively. Such modulators have gone out of fashion, in part because they were insufficiently robust, and in part because of their inability to trap highly volatile analytes, despite using modulation capillaries with much thicker stationary‐phase films than the ^1^D column (“phase‐ratio focusing”). Thermal modulators using a coolant, such as carbon dioxide or, more commonly, (evaporated) liquid nitrogen, have proven extremely efficient in creating narrow, concentrated ^2^D injection pulses, even for highly volatile analytes [23, 24]. The main disadvantage is the need for a coolant—and the associated costs. In the case of liquid‐nitrogen‐cooled dual‐stage modulation, these have been estimated at 150€ per instrument per day. Although such a number can be influenced by numerous factors, it is clear that consumable costs can be considerable. This has been the main driver for the development of a new generation of “coolant‐free” or “consumables‐free” thermal modulators. Unlike the earliest modulators, cooling is used in these new devices, but the method of cooling is different. In refrigerator‐type cooling, a coolant is used, but in a closed loop, so that, in principle, no consumables are required. Thermo‐electric cooling using Peltier elements does not require any coolant.

In Figure 1, we have classified valve‐based modulators under flow modulators, because valves serve to direct or diversify the flows of carrier or auxiliary gases. In comprehensive LC × LC thermal modulation is barely used [25], because the effect of temperature on retention is too small (except for high‐molecular‐weight analytes [26]). Valve‐based modulators are almost exclusively used in LC × LC. In contrast, valves in the mobile‐phase flow path are scarcely used in GC × GC, in spite of early successes [3, 27]. This is due to several significant disadvantages, which include (i) the need to operate the valve continuously at a high temperature, (ii) the risk of analyte adsorption or (catalytic) degradation involving valve materials, and (iii) broader peaks and more tailing in comparison with thermal modulators due to the hydrodynamics of emptying a loop and due to the required connections. This latter effect may be mitigated to some extent by a high ^2^D flow rate, but this reduces the chromatographic efficiency and detector compatibility of the GC × GC system.

Instead of using sampling valves, valves may be positioned outside the flow path, and differences in pressure may be used to control flows and to modulate the effluent from the ^1^D column. Inert glass or micromachined 3‐port or 4‐port GC splitters, or commercially available Deans’ switch devices [28], may be used to minimize the risk of adsorption. This may mitigate some of the above disadvantages, but one that pertains is the absence of inherent analyte focusing. This tends to result in a much lower sensitivity (*S/N*) than what can be achieved with thermal modulation, especially when low duty cycles (small fraction of ^1^D effluent sent to the ^2^D column) are encountered, as with diverted‐flow modulation (see below). Significant advantages are (i) the inherent simplicity of flow‐modulation devices, (ii) the fact that no consumables (coolants) are required, and (iii) the universal applicability. Flow modulation is neither challenged at the high‐volatility end (no need to trap), nor at the low‐volatility end (no need to remobilize). Thus, it is understandable that flow modulation keeps receiving significant attention.

### 2.1 Progress in thermal modulation

Considerable effort has been devoted to the development and evaluation of coolant‐free (or “cryogen‐free”) thermal modulation. Mucédola et al. [29] described a simple thermal modulation based on similar resistive‐heating principles as the original Liu and Phillips modulator [9]. The authors used a 1.0 m × 250 μm id MTX‐5 metallic modulation loop with a thick (0.50 μm) film of stationary phase, installed between a 250‐μm id ^1^D column and a 100‐μm id ^2^D column. Trapping took place within the GC oven, so that it relied on phase‐ratio focusing. The first and last 200 mm of a 1 m modulation loop were resistively heated during periods of 355 or 500 ms, depending on the power supply. A quasi‐stop‐flow regime that helped avoid analyte breakthrough was achieved because the heat pulses resulted in a temporary increase in gas viscosity, resulting in a decrease in flow rate. Muscalu et al. [21] described a single‐stage modulator that also relied on resistive heating of a trapping capillary. This coated metal capillary was clamped between two ceramic blocks used for cooling to improve the trapping of volatile analytes. The authors used a micro‐electron‐capture detector (μECD) for the analysis of chlorinated compounds. The study was focused on the precision (repeatability) of the measurements and the consumable‐free system was found to perform similarly to cryogenic modulators using liquid nitrogen. Jacobs et al. [30] tested such a single‐stage modulator successfully. They recommended that the simple and compact design of this consumable‐free modulator could make it amenable with portable GC instruments.

The commercial Zoex ZX2 modulator uses a refrigerator unit to cool nitrogen down to −90°C (and, hence, should not be called a cryogenic modulator). Wilde and Rowland [31] used GC × GC‐MS with a refrigerator‐type modulator to identify the structures of a large number of naphthenic acids. They used a 250‐μm id ^1^D column, a 100‐μm id modulation capillary, and a 100‐μm id ^2^D column, and tested modulation periods of 2, 4, and 6 months, with longer times not resulting in more identifications. The temperature of the hot jet used during the release stage was programmed and kept well above the temperature of the ^1^D oven. The temperature of the ^2^D oven was kept 40°C above the ^1^D one. Alam et al. [32] used the same type of modulator and ^1^D and ^2^D columns with the same id in trying to identify hydrocarbon isomers with GC × GC‐MS. Giri et al. [33] also used several column sets with 250‐μm id ^1^D columns and 100‐μm id ^2^D columns in studying hydrocarbons and various other classes of compounds with GC × GC‐MS. Robson et al. [34] used the refrigerator‐type modulator in a very extensive study into the composition of oil samples. After extensive group‐type preseparations (and other methods of characterization), they subjected the fractions obtained to GC ×0020GC‐MS. Because the ^2^D column had an id of 250 μm (same as the ^1^D column) and because an injection (release) pulse of 400 ms was used, the performance of the modulator was not really challenged. All these studies used high data acquisition rate TOF MS instruments, underlining their increasing value in combination with GC × GC separation. Different ionization techniques and conditions are employed. Alam et al. [32] used electron‐ionization at different energies, and Giri et al. [33] used photoionization, all to obtain additional structural information including molecular ions. Al‐Rabiah et al. [35] used the ZX2 modulator in studying sulfur speciation in oil products. The exit of the 2D column was connected to two detectors, an FID and an SCD, in series. They identify a minimal modulation time of 1 s and a restriction to analytes with a volatility lower than or equal to that of C_7_ alkanes as disadvantages of the modulator.

Luong et al. [36] tested a solid‐state modulator (SSM1800, commercially offered by J&X Technologies), which used thermoelectric cooling to achieve an operating range between −50°C and 50°C. “Mica‐thermic heating” up to 350°C is used for zones at the entry and exit of the modulation capillary. Indirect heating through a mica stone is energy effective, but does not allow rapid changes in temperature. The modulation capillary is interchangeable and dedicated to a specific range of analytes (in terms of volatility or carbon numbers of *n*‐alkanes). The exact nature of the stationary phase in the modulator columns is not disclosed by the manufacturer, but a retentive film, probably with a large thickness, is needed to apply the system to highly volatile analytes (down to C_2_ hydrocarbons, as claimed by the manufacturer). High temperatures in the heated zones and a high release temperature may be detrimental to the stability and robustness of the modulator. The modulation capillary, which is located outside the GC oven, moves back and forth through the heated and cooled (center) zones to achieve the dual‐stage thermal modulation. The instrument also has a provision for an auxiliary gas flow at the modulation stage, so that faster release of analytes can be achieved [37], so that it may also be classified as a hybrid modulator. Boswell et al. [38] compared the performance of the SSM with conventional coolant‐driven modulation for bitumen samples, using a constant mass‐flow range through the two (^1^D and ^2^D) columns, both with an id of 250 μm. They found a similar performance for the two types of modulators. Giocastro et al. [39] studied modulation capillaries for use in the SSM. They found capillaries with a 180‐μm id (×0.18 μm film thickness) preferable to those with a 250‐μm id (×0.25 μm film thickness), as the former yielded narrower injection pulses into the ^2^D column.

### 2.2 Progress in flow modulation

In flow modulation no focusing effect can be achieved by creating a cold spot in the flow path of the ^1^D effluent. Yet, very narrow injection bands need to be created for injection on the ^2^D column, so as to achieve GC × GC operation. There are basically two ways to achieve this: (i) collecting small fractions of the ^1^D effluent and discarding the remainder to waste (“flow‐diversion” modulators), or (ii) diluting fractions of the ^1^D effluent by mixing these with an additional high flow of carrier gas (“differential‐flow” modulators). The first type of operation is characterized by a low duty cycle (only a small fraction of the analytes is passed to the ^2^D column) and, as a consequence, low analytical sensitivity. The second option may result in duty cycles of (almost) 1, but is characterized by high ^2^D flow rates. The sensitivity is not affected if a mass‐flow‐sensitive detector, such as an FID, is used. High ^2^D flow rates are generally not compatible with MS detectors. The Mondello group [40, 41, 42] has made significant efforts to alleviate this problem. The high flow rates also put restrictions on the column dimensions. Narrow columns are not compatible with the high flow rates, as this would lead to excessively high pressures. As a result, the efficiency and peak capacity that can be attained in the second dimension are restricted.

Fast‐switching, pneumatically activated diaphragm valves were used successfully in early GC × GC studies. Bruckner et al. [3] achieved 50‐ms‐long ^2^D injection pulses, roughly equal to the injection band width reported for thermal modulation [43]. Mohler et al. [44] developed a modulator with a single six‐port valve that yielded a duty cycle close to one. Instead of flushing the modulation loop with ^1^D effluent to waste, the exit of the loop was plugged during the load cycle. A high ^1^D pressure meant that an aliquot of sample was compressed in the loop. Upon switching, this fraction was injected into the ^2^D column. A relatively large ^2^D column was used (3 m length, 250 μm id, 0.25 μm film thickness) to accommodate a high ^2^D flow rate, while maintaining a pressure below that of the 1D column, needed to achieve compression in the loop.

Cai and Stearns [45] describe a modulator based on a simple six‐port valve that is configured such that (i) in the loading position the ^1^D and ^2^D columns are connected in series, so that a fraction of the ^1^D effluent is loaded on the ^2^D column, rather than in a fraction‐collection loop, and (ii) in the hold position the flow in the ^1^D is temporarily reversed and then stopped, while the ^2^D separation is performed. The auxiliary gas flow is not needed when the inlet pressure of the ^2^D separation is equal to that when the two columns are connected in series. The stop‐flow operation of the ^1^D column allows the use of long secondary columns at the expense of longer total analysis times. ^2^D columns up to 30 m in length (with 250 μm id) were studied. Some loss in the ^1^D separation was observed. The authors signaled the ease of switching between conventional 1DGC operation (with the two columns in series) and GC × GC operation as an advantage.

In a differential‐flow modulator, a modulation capillary is filled slowly with the effluent from the ^1^D column, after which it is emptied rapidly at a much higher flow rate into the ^2^D column. Griffith et al. [46] demonstrated a more efficient flushing of the modulation capillary in backflush mode. This was apparent from the return to baseline for high‐concentration analytes. Breakthrough of the ^1^D effluent into the ^2^D column in case the modulation capillary is overfilled is avoided by installing a vent restrictor capillary that decouples the modulation capillary from the ^2^D column during the fill cycle. Giardina et al. [47] developed an accurate model for the design and operation of such a backflush or “reverse fill/flush” (RFF) modulator. Moreira de Oliveira et al. [48] compared optimized forward‐flush (FF) and RFF modulators and found the latter to perform best for C_20_^+^ hydrocarbons.

Seeley et al. [49], a group who has been instrumental in developing flow modulators over the years [27, 28, 50, 51], describe a simple modulation device that can be used either in flow‐diversion or differential‐flow mode. They describe their modulator as the GC × GC equivalent of a split or splitless injector for 1DGC. Such a modulator was said to allow precise control over the width of the ^2^D injection pulse without altering the flows in the ^1^D and ^2^D columns. It was used for a detailed study on the width of ^2^D injection pulses produced by the modulator [52]. The recommendation from this study was that the optimum pulse width is of the order of the minimum band width that can be produced by the ^2^D separation. Ghosh et al. [53] constructed and optimized a Deans’ switch from readily available components that allowed them to achieve very narrow (<50 ms) ^2^D injection pulses at a moderate ^2^D flow rate (2 mL/min) that is compatible with a wide range of GC detectors. The main disadvantage of this modulator was its low duty cycle.

In pulse modulation [54], an auxiliary gas flow after the ^1^D column is created to achieve a temporary increase (negative‐pulse mode) or decrease (positive‐pulse mode or “vacancy chromatography” mode) of the analyte concentrations, which results in a short ^2^D chromatogram on top of (or underneath) the relatively slow‐moving ^1^D chromatogram. Both the 1‐D chromatogram produced by the ^1^D column and the 2‐D chromatogram can be mathematically derived from the resulting signal. A pulsed modulator is quite simple to construct and may yield sharp ^2^D injection pulses, but the sensitivity is reduced, because only differences in concentration are detected, instead of absolute concentrations. In recent years, the idea of pulsed modulation has been revisited and built upon. First, Freye et al. greatly reduced the pulse length for the ^2^D injection, down from about 500 to 50 ms [55]. Trinklein et al. [56] introduced “dynamic pressure gradient modulation” (DPGM). With a similarly simple setup as Cai an Stearns [54], they managed to essentially stop the ^1^D flow during most of the modulation time of 750 ms in the example of the paper and then only allow very short (60 ms) pulses of ^1^D effluent to enter the ^2^D column. An oscillating pressure gradient was created, which explains the name given to the technique. Because all of the ^1^D effluent eventually reaches the detector (ie, a duty cycle of 1), the approach was referred to as a “full‐modulation approach.” Schöneich et al. [57] demonstrated the validity of the DPGM approach and its compatibility with TOF MS detection. They found a 10‐ to 20‐fold increase in signal intensity and a fivefold increase in *S/N* ratios relative to unmodulated peaks. Although the timing is much faster, the stop‐flow DPGM approach is reminiscent of the stop‐flow modulator [proposed by Harynuk and Gorecki in 2004 [58].

Guan *et al.* [59] describe a simple modulator that employs a solenoid valve (outside the column oven) that connects the carrier‐gas supply line with a T‐piece positioned between the ^1^D and ^2^D columns (inside the oven). This modulator allowed three different modes of operation: bypass stop‐flow (closing one port of the solenoid valve), vent stop‐flow (opening one port of the solenoid valve to atmosphere), and quasi‐stop flow. The best results were obtained in the latter mode, generating injection pulses as narrow as 34 ms. To realize this, the authors needed to optimize flow restrictions and internal volumes. The high ^2^D flow rate forces the authors to use a rather large ^2^D column (3 m × 220 μm id). The authors suggest that their modulator performs similarly to the DPGM approach described above, while being much simpler.

Bahaghighat et al. [60] used pulse modulation to achieve “ultra‐narrow” differential‐concentration peaks for use in GC × GC and comprehensive three‐dimensional GC (GC × GC × GC or GC^3^). The first modulator yields ^2^D chromatograms on top of the ^1^D signal, and the second modulation creates a third dimension (^3^D) on top of the already complex ^2^D signal, creating great challenges for data analysis. The authors demonstrated GC^3^ analysis with analysis times of ^1^ *t*_anal_ = 12 min, ^2^ *t*_anal_ = 1.2 s, and ^3^ *t*_anal_ = 60 ms in the respective dimensions. Gough et al. [61] studied optimal column design for GC × GC and GC^3^. Focusing on the phase ratio (*β*), which in GC is defined as:

$$\beta \;=\frac{V_{m}}{V_{s}}\;\approx \frac{d_{c}}{4d_{f}},$$

where *V_m_* is the total volume of mobile phase in the column, *V_S_* is the total volume of stationary phase, *d_c_* is the column diameter, and *d_f_* is the film thickness. Typical values of the diffusion coefficient of the analyte in the mobile phase (carrier gas) and the (silicone) stationary phase result in a typical *β* value of 250 (*d_f_* = 0.001*d_c_*, corresponding to an optimal reduced film thickness *δ_f_* of 0.3 [62]). Gough et al. arrived at a much lower phase ratio for their ^1^D column (^1^ *d_c_* = 180 μm, ^1^ *d_f_* = 0.4 μm, ^1^ *β* = 112.5), while using a conventional film thickness in their ^2^D and ^3^D columns (^2^ *d_c_* = ^3^ *d_c_* = 100 μm, ^2^ *d_f_* = ^3^ *d_f_* = 0.1 μm, ^2^ *β* = ^3^ *β* = 250). They computed a peak capacity of 30,000 [61] for their GC^3^ system. In another paper, they used a 40‐m long, apolar (DB‐5) ^1^D column, followed by a 2.5‐m long medium‐polar (RTX‐200) ^2^D column, and a 1‐m long polar (DB‐HeavyWax) ^3^D column. All three columns had an id of 180 μm and stationary‐phase film thicknesses of 0.18 μm, resulting in phase ratios of 250. This system was coupled to a ToF MS detector and yielded an overall peak capacity of 35,000 [63].

Tungkijanansin and Kulsing [64] described an ingenious approach in which they split and recombine each fraction of the ^1^D effluent into several (two or four) peaks with different volumes (delays), but no different pressures, between the different lines. The resulting pattern of a single peak is akin to that of modulation, except that the areas of all the modulated signals from one fraction are about equal. The authors speak of “Splitter‐based Non‐cryogenic Artificial Trapping” (SNAT). From the total chromatogram with regular patterns, a modulated chromatogram can be deduced. No coolants are required, but the system is quite complex, and the analyte concentrations are lowered prior to injection in the ^2^D column.

## 3 DEVELOPMENTS IN GC × GC DETECTION

Major developments in the area of detection for GC × GC have been scarce in the past decade, however, some incremental improvements to existing detection techniques, as well as, the importance of detection to the understanding of the field, warrant a closer look at this subject.

In early GC × GC, the FID was the obvious first choice. The many advantages of the FID as a near‐universal detector that offers very low detection limits and a linear working range spanning many decades were as relevant for GC × GC as they were for 1DGC. The only adaptation that was needed was to modify the electronics to allow for a high sampling rate and a short effective time constant. In addition, the main application fields of the early GC × GC work were oil and (petro‐)chemical samples, which are highly suitable for fingerprinting by GC × GC. GC × GC‐FID yielded structured chromatograms that offered highly robust fingerprints and excellent options for quantitation [65, 66, 67, 68, 69], often of classes (group types) of analytes. However, the FID did not offer any qualitative information on the analytes. Identification of analytes was largely based on the injection of known standards and reasoning (largely interpolation and extrapolation) to assign other peaks.

In terms of universal detection methods, there have not been many alternatives to the FID. The helium ionization detector or barrier discharge ionization detector has been tested as an alternative for FID in the context of (low‐)flow modulated GC × GC [70]. It offers greater sensitivity at the expense of a narrower dynamic range, without increasing the extra‐column band broadening, when tested with various saturated and nonsaturated hydrocarbon mixtures. This makes it a possible alternative for FID in low‐flow applications. Important to note, however, is that this detector does not respond in a linear fashion to the mass of carbon, thus, not allowing quantitative analysis without external calibration. The latter is one of the strong suits of FID detection.

Element‐specific detectors have been used and are being used in combination with GC × GC. A classic example is the study the effects of hydrotreating and other desulfurization processes on the composition of oil products [71, 72, 73]. An SCD detector responds specifically to sulfur compounds and provides very low detection limits. GC × GC‐SCD has recently been used, often in combination with GC × GC‐MS, for highly diverse applications, including the detection of sulfur compounds in pyrolysis oils obtained from lignin biomass [74] or from plastic waste [75], the identification of sulfur compounds in aroma of wine [76] or Chinese liquor (*Laobaigan Baijiu*) [77], and in cannabis [78]. Djokic et al. [79] used GC × GC‐SCD to study the effects of sulfur compounds on the steam‐cracking process. Likewise, a nitrogen‐chemiluminescence detector can be used to specifically detect nitrogen‐containing compounds in 2‐D chromatograms. Recent examples include nitrogen compounds in mineral‐oil products [18, 80] pyrolysis oils obtained from microalgae [81] or plastic waste [75], and nitrogen compounds in cigarette smoke [82].

A FPD is neither as specific, nor offers such low detection limits for sulfur compounds as an SCD detector. However, it is a much simpler and more robust device. It has been used in combination with GC × GC to characterize jet‐fuel samples [83]. In phosphorous mode, it has been used to study pesticides [84]. Electron‐capture detectors are selective for halogenated compounds, and they have also been found to be useful in combination with GC × GC. Examples include the detection of brominated disinfection by‐products [85], polychlorinated biphenyls in insulating oils [86], chlorinated paraffins in sediments [87], and, most prolifically, halogenated pollutants in water samples [88, 89, 90]. Atomic‐emission detection (AED) allows tuning to a variety of elements and groups of elements simultaneously. It has been used in combination with GC × GC [91, 92, 93], but the last published report on the use of GC × GC‐AED dates to 2005. Similarly, the use of a nitrogen phosphorus (thermionic) detector, which is sensitive, but not stable in its response, in combination with GC × GC has not been reported on since 2008 [94, 95].

Predictably, after the GC × GC technique itself started to become robust and established, MS detection soon became vitally important. In the decade (2000‐2010), after the first reports that involved online GC × GC‐MS by Frysinger and Gaines (1999) [96] and by van Deursen et al. (2000) [97], Scopus reveals that 54% of all GC × GC papers mentioned MS. Post 2010, this number has risen to 78%. Although not all reports concern online GC × GC‐MS, the overwhelming importance of MS detection is evident.

Most GC × GC systems can be readily coupled with MS instruments. For some modulators (eg, differential flow modulation), hyphenation may be more challenging. In such cases, a frequently utilized solution involves splitting after the ^2^D column. This allows a portion of the effluent to be directed towards the MS, while another portion is directed to waste or to another detector, typically an FID [98, 99, 100, 101]. Such setups capitalize on the quantitative capabilities of the FID and combine this with the qualitative strengths of MS. Bauwens et al. [102] coupled a liquid‐chromatographic (LC) preseparation online with GC × GC with dual detection, resulting in a powerful LC‐GC × GC‐FID/MS platform. Zanella et al. [103] described a GC × GC platform with parallel MS and vacuum UV (VUV) detectors. This combination offers exciting prospects for the identification of isomeric compounds. However, large libraries of UV spectra to augment those of MS spectra are not yet available. Similar to GC × GC as a whole, the demand for data analysis tools has increased in recent years [104].

The most important requirement for the MS instrument is a very high scanning rate, to sample each ^2^D peak with a sufficiently high number of spectra. The sampling rate is mainly determined by the choice of MS. It was instantly recognized [97] that ToF‐MS instruments are the obvious first choice, because of the very high data acquisition rate possible (up to 100 Hz) and because all ions in one spectrum are sampled at the same time. Because of their greater availability, quadrupole MS (Q‐MS) instruments were seriously tested in some early studies [105, 106, 107, 108, 109, 110], but they are inherently much slower. Raising the acquisition rate comes at the cost of reduced *S/N* ratios and mass‐spectral resolution. The scan range can also be narrowed to improve the acquisition rate. When a scan is taken on the slope of the peak, the concentration of the analyte will vary, resulting in distorted and varying spectra. Rapid‐scanning Q‐MS systems have become available, but they are only occasionally used in contemporary GC × GC‐MS practice [111, 112, 113, 114, 115]. Triple‐quadrupole (QqQ) instruments have been demonstrated for online use with GC × GC [116, 117, 118]. Such systems allow quantitative MS/MS measurements of target analytes, which enhances the specificity and lowers the detection limits. When looking for selected primary ions and product ions (during selected time intervals), the scanning rate of the MS instrument is not a critical factor. Hybrid quadrupole—TOF (Q‐TOF) instruments offer all the advantages of QqQ instruments, plus a much higher mass resolution. Also, they can be used as regular fast‐scanning ToF instruments. Therefore, Q‐TOF instruments are increasingly used in combination with GC × GC [119, 120].

Orbitrap‐MS instruments were recently also online, coupled with GC × GC. Crucello et al. [99] describe a combination of a flow‐modulated GC × GC instrument with an Orbitrap MS for determining naphthenic acids in water samples (after extensive sample preparation). A 20 m long, 180 μm id, 0.18 μm film thickness ^1^D column and a 2.5 m × 250 μm id × 0.25 μm ^2^D column were used, with an auxiliary helium flow augmenting the carrier‐gas flow during modulation. Passive splitting was employed after the ^2^D column, with about one‐third of the total flow entering the MS. The Orbitrap scan range was 50–600 Da with a scan rate of about 25 Hz. Under these conditions, the MS resolution (Δ*m*/*m*) was 15,000 at a mass‐to‐charge ratio (*m*/*e*) of 200.

The proliferation of advanced MS systems for use in combination with GC × GC has also led to the use of other ionization techniques than the 70‐eV electron ionization (EI) that is the benchmark method in GC‐MS. Giri et al. compared EI, photo ionization (PI), chemical ionization (CI), and field ionization (FI) of GC × GC‐MS for a heavy oil product [121]. All the latter three soft‐ionization techniques yield better molecular‐ion information than EI (except for polycyclic naphthenic compounds). CI is highly selective and, therefore, less compatible with comprehensive fingerprinting by GC × GC‐MS.

Soft ionization methods after GC × GC separation are especially relevant in combination with HR MS and MS/MS techniques [122, 123]. GC × GC‐PI‐HRMS has been pioneered by the Zimmerman group [124, 125]. Giri et al. [33] used PI in combination with HR ToF‐MS. Genuit and Chaabani [126] and Qian and Wang [127] have coupled GC × GC with FI‐MS. Tranchida et al. [128] studied EI with lower energies, such as 20 eV, as a means to achieve softer ionization. However, 70‐eV EI was still used in GC × GC‐QqQMS studies [116, 117, 118].

## 4 RETENTION MODELLING

It cannot be denied that GC × GC is more complex than 1DGC. The number of relevant parameters that need to be selected or optimized when developing a GC × GC method is more than twice that of GC [129]. The plethora of possible combinations of realistic values of relevant parameters makes method development a difficult, if not daunting task. One way to reduce the task is by eliminating parameters that are thought to be of little impact in the context of the application. If the analytes are a priori known, predicting their elution time may reduce method development efforts. Different retention‐time or retention‐index models have been proposed to predict the elution time of analytes from the GC × GC separation. However, the large number of mutually dependent parameters makes it quite intricate to develop adequate models [129].

The challenge of retention modeling in GC × GC can be divided in the two main tasks: (i) determining the analyte‐independent pressure and flow characteristics in the two columns and (ii) establishing analyte‐specific retention parameters. The first challenge is especially significant in thermal modulation. From knowledge of the lengths and diameters of the ^1^D and ^2^D columns, the inlet and outlet pressure, and the viscosity of the gas, the pressure, and linear velocity at any point can be calculated from chromatographic theory [130] or through an iterative process [131]. When temperature programming is applied, as is almost always the case in GC × GC, at least for the ^1^D separation, the linear velocity will vary, either because the gas viscosity varies with time (constant‐pressure conditions) [132] or because of thermal expansion (constant‐flow conditions). The main complication arises from the effects of the thermal modulation on the flow characteristics. The variation in temperature creates an additional time‐variant effect that complicates the modeling. Any uncertainty in these calculations is propagated in the model, leading to increased errors. The second challenge is typically expressed in terms of thermodynamic parameters, that is, the partial molar enthalpy and entropy of transfer of the analyte between the mobile phase and the stationary phase and its molar heat capacity at constant pressure. These latter parameters are typically obtained from 1‐ or 2‐D experiments on columns with the same stationary phase [133, 134, 135], because no accurate, generally applicable predictive model exists for the purpose.

Predicting ^2^D retention times is thought to be more challenging than predicting 1D retention times [136, 137]. In part, this is due to variations introduced by the modulator, which are not accounted for in the retention models. Predicting retention times is further complicated if MS detection is creating vacuum conditions at the outlet of the ^2^D column. Jaramillo and Dorman [138] studied the prediction errors in ^2^ *t_R_* values and they derived an empirical correction, based on a polynomial fit to the data obtained for alkanes. They later described an easier way to obtain retention data for comparison and identification purposes [139].

Gaida et al. [136] considered the two underlying 1DGC systems individually to predict GC × GC behavior in what they call a “top‐down” approach. The modulator was considered an injection device for the ^2^D separation. The latter was treated as isothermal, given the short duration and the programmed oven heating rate, taking into account a constant mass flow and vacuum conditions created by MS detection.

## 5 DATA ANALYSIS

Although the added separation power of GC × GC significantly reduces the likelihood of co‐elution, it is unlikely to separate all peaks of interest in a complex mixture. Thus, GC × GC does not readily provide the characteristics (peak position, height, area, width symmetry, etc.) of peaks of interest. Innovative approaches are particularly required when multichannel detectors are employed because the wealth of information contained in the large multidimensional datasets obtained cannot be harvested with existing tools. It is, thus, not surprising that data processing continues to receive a lot of interest. The subject has been extensively covered by several excellent recent reviews [140, 141, 142, 143].

The present review will instead provide a general picture of the data‐analytics workflow and clarify the most important concepts to emphasize the importance of data processing, pointing out recent developments where relevant.

**FIGURE 2:**
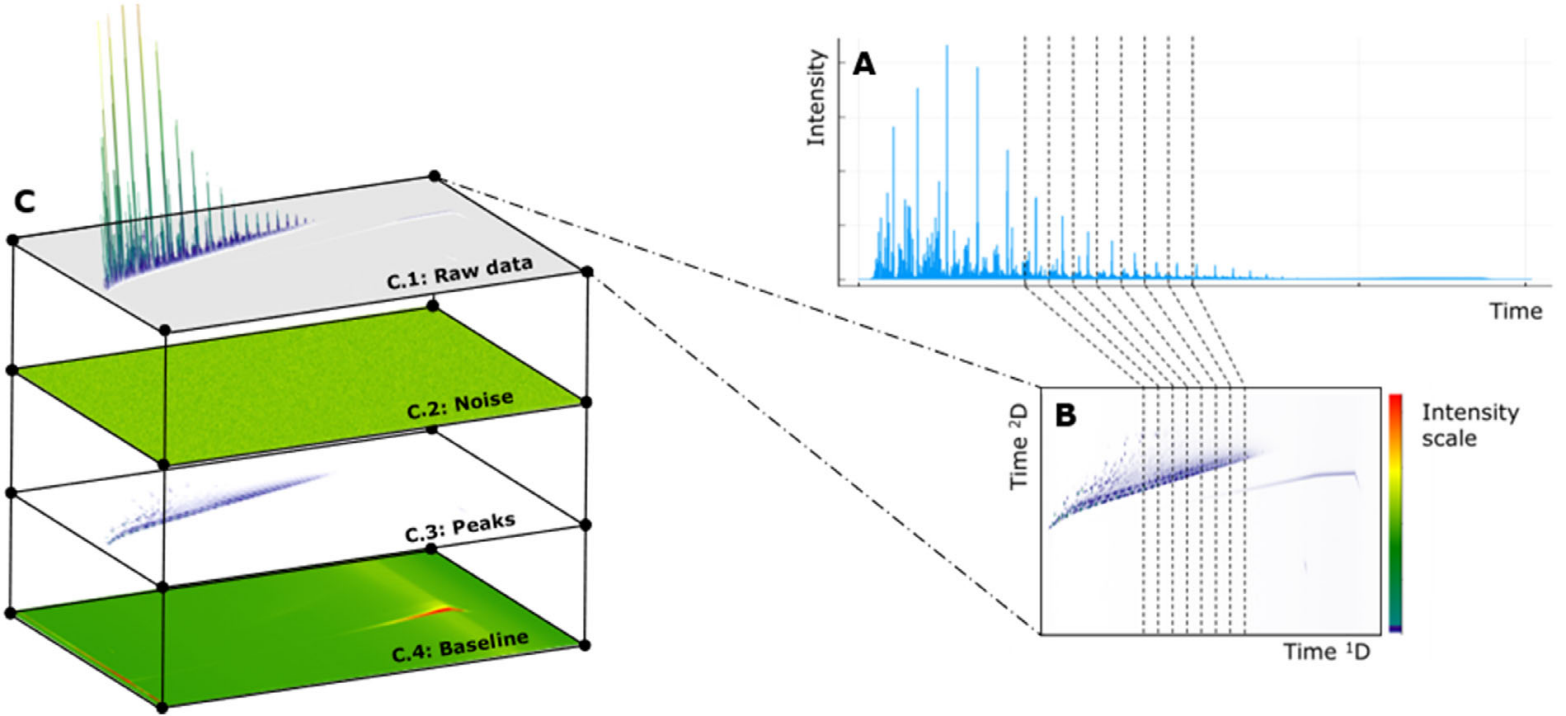
Structure of GC × GC chromatogram. (A) Raw signal of a GC × GC chromatogram. (B) Folded GC × GC chromatogram, in which each of the individual modulations is depicted by a dotted line. (C) Decomposed chromatogram with (C1) the raw data, (C2) the high frequency, noise component, (C3) the medium‐frequency chromatographic peaks, and (C4) the low‐frequency baseline drift. Simulated data based on experimental profiles.

To understand the challenges of data analysis for GC × GC, it is useful to understand the structure of a GC × GC chromatogram. The commonly known GC × GC chromatogram in the form of a heatmap or contour plot (Figure 2B) is not actually how the instrument records the data. The modulator facilitates the comprehensive transfer of analytes from the typically long (≥ 10 m) ^1^D column, to the shorter ^2^D column (typically ≤ 2 m). This ^2^D column terminates at the only detection point in the chromatographic system. As there is only one detector for both chromatographic dimensions, the resulting raw data is a 1‐D time versus intensity trace (Figure 2A).

The familiar heatmap‐style plots (or color plots), with the ^1^D retention time on the x‐axis, the ^2^D retention time on the y‐axis, and color denoting intensity, are simply a different visual representation of the aforementioned 1‐D raw data trace. The 2D heatmap can be created through a process referred to as folding, where the chromatogram is cut into sections the length of the ^2^D analysis time (or the modulation time). Each of these sections becomes a new column in a data matrix, resulting in a rectangle of data, with ^2^D chromatograms along the vertical axis and the virtual ^1^D separation now emerging along the horizontal axis [144, 145]. Incorporating a multichannel detector (eg, MS or VUV) increases the complexity of the data by adding another data dimension [145, 146, 147]. Due to the higher dimensionality of the data, manual processing is extremely challenging [148].

The type of data‐analysis workflow is largely dictated by whether the analytical method concerns targeted or nontargeted analysis. Targeted analysis primarily concentrates on known compounds, aiming to verify their presence or determine their concentration in a sample. On the other hand, untargeted analysis is an increasingly significant area of research that aims to identify and understand the compounds present in a sample without any prior knowledge of their existence or identity [143] Targeted analysis is relatively straightforward as it involves prior knowledge of specific compounds, which simplifies their identification and quantification. Recent research in the context of GC×GC mainly focuses on untargeted screening.

### 5.1 Signal preprocessing: The need for a common dataset

Any chromatogram, or analytical signal, can be divided into several components: low, medium, and high frequency one. For chromatograms, these would concern the baseline (Figure 2‐C.4) drift, chromatographic peaks (Figure 2‐C.3) and noise (Figure 2‐C.2), respectively.

The first step in a data‐analysis workflow concerns signal preprocessing with the aim to remove the high‐ and low‐frequency components from the signal, and thus, exclusively yielding the chromatographic peaks. This is also known as baseline correction, where the undesired signal contributions that arise from variations in oven temperature, column degradation, and low‐frequency detector noise (background) are removed [143, 145]. For the multichannel data arising from GC × GC‐MS, several strategies exist, which have been extensively reviewed elsewhere [146].

One of the most elementary signal preprocessing techniques concerns the removal of the high‐frequency noise to improve the *S/N* ratio. A very common general approach for this purpose in chromatography is the Savitzky‐Golay filter [149, 150]. In contrast to baseline‐correction methods, very little has changed in the way of smoothing algorithms in the last few years.

Baseline‐correction models can be mainly categorized into two groups, that is, parametric and nonparametric [149, 151]. Parametric approaches fit a polynomial to the baseline and subtract its contribution from the raw data [152, 153], therefore, assuming that the baseline is of a certain (predefined) form that can be captured by the employed baseline model [154] Nonparametric approaches are not based on assumptions prior to (typically) applying multivariate resolution methods [149, 151].

Due to the fact that GC × GC chromatograms are in fact long 1D signals, baseline‐correction approaches designed specifically for 1D chromatography are also of interest. Several such approaches were published in the last decade including several types of penalized‐least‐squares methods [155, 156], corner‐cutting [157], baseline estimation and denoising using sparsity [158], wavelet‐based methods [159], and the newer neural network [160, 161] approaches.

One challenge commonly faced in the development of signal‐processing tools is the numerical evaluation of their performance. At this point, it is important to note that chemometric tools can never be perfect and will induce errors. For example, suppose we have a peak with an unknown area on top of a signal with baseline drift and noise. We could now employ a baseline correction algorithm to distil the peak from the rest of the signal. However, to assess whether the resulting value is correct, we would need to know the original area of the peak, which we, of course, do not. To avoid this chicken‐and‐egg problem, authors are increasingly working with simulated datasets to test their algorithms. The advantage is that the peak characteristics are known for these simulated signals. However, the simulated signals are often simpler than real data and typically do not capture experimental detail such as peak asymmetry, peak coverage, baseline drift, and noise level. Other groups, therefore, opt to use experimental data and settle for relative comparisons.

To address this challenge, Niezen et al. developed a tool to generate 1D chromatographic signals with a high degree of reality, superimposing noise levels and collecting baseline‐drift signals on distributions of peaks that were modeled after experimental chromatograms [162]. Using these simulated, yet near‐realistic chromatograms, Niezen et al. computationally reviewed a number of background‐correction tools. The authors found that the performance of each investigated algorithm relied heavily on signal characteristics (noise, peak coverage, etc.). For example, some algorithms withstood relatively high noise levels better, whereas other coped better with denser distributions of peaks.

One example of a recent development of a tailored baseline correction method is that of Mikaliunaite et al. The authors designed their strategy to accommodate the significant baseline disturbance encountered in dynamic‐pressure‐gradient (flow) modulation [153].

**FIGURE 3:**
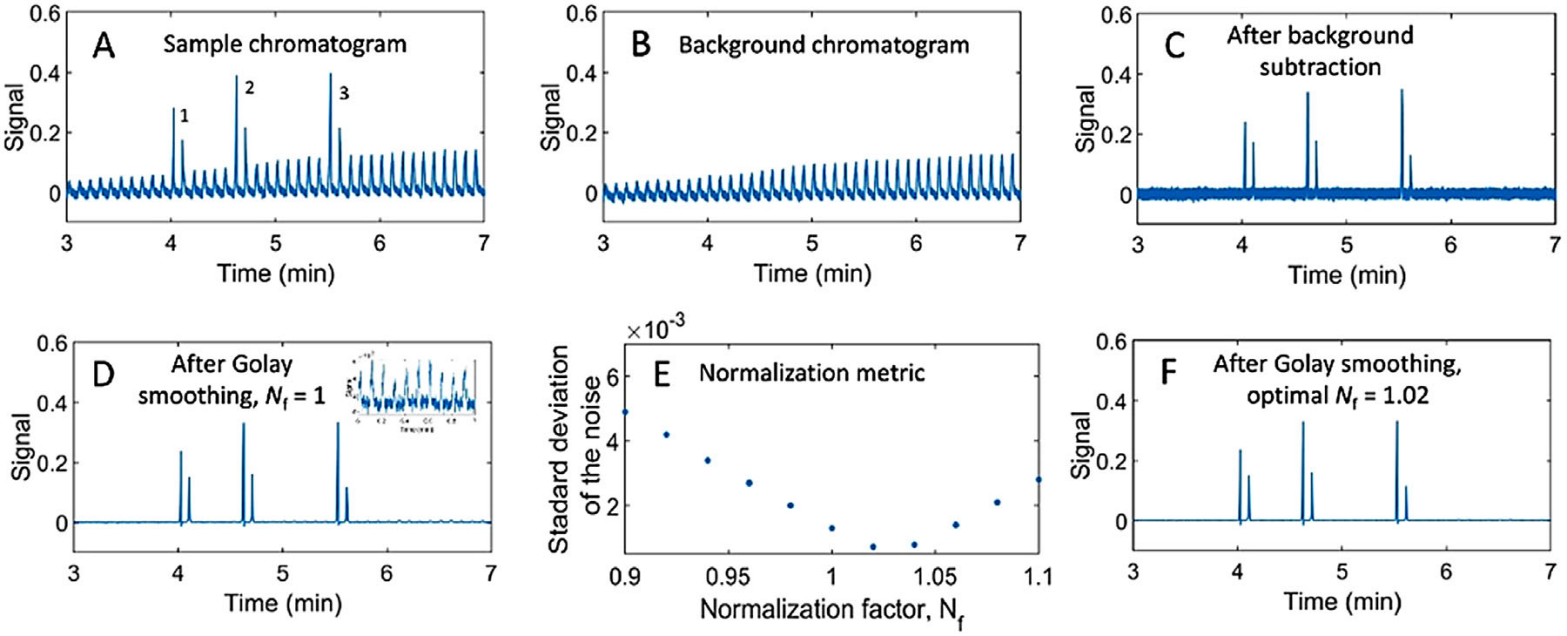
Example of the background‐correction strategy designed for dynamic‐pressure‐gradient (flow) modulation. Panel A shows a sample chromatogram, B represents a background chromatogram, which is subtracted from the signal in panel A; it is the result is presented in C. After smoothning using a Savitzky‐Golay filter, the signal shown in D is obtained. Panel E displays the employed normalization metric to determine the optimal normalization factor, resulting in the signal shown in panel F. Reproduced from [153] with permission.

In Figure 3 [153], an example of the workflow is presented. The pressure oscillation is clearly visible in the chromatograms of both samples and blanks (Figure 3A and B). Therefore, blank (Figure 3B) subtraction was performed on the original chromatogram (Figure 3A). This resulted in a chromatogram with a relatively noisy baseline (Figure 3C) to which a Savitzky‐Golay filter was applied to take out the high‐frequency noise (Figure 3D). Thereafter, still a rhythmic baseline disturbance can be seen. Therefore, optimal normalization conditions were determined (Figure 3E) and applied (Figure 3F).

Once signal preprocessing has been completed, the next step is to consider data preprocessing. The objective of data preprocessing in GC × GC is to remove any retention time shifts that may occur in replicate injections, causing potential confusion in the analysis. Such chromatographic variations may be caused by fluctuations of carrier‐gas pressure, temperature, column degradation, or maintenance. Data alignment can correct for these retention‐time shifts. Manual alignment can be performed using peak tables, but this is a laborious task [145]. Weggler et al. conducted a comparison of several automated data‐alignment tools and found their performance to be similar. It is crucial to maintain consistency in alignment, regardless of the approach taken, particularly when using additional statistical methods in further research [163].

While commercial support of data‐analysis strategies has been extremely useful to advance the proliferation of multidimensional 2D chromatography, one unfortunate issue is that many of the commercial algorithms are essentially “black box” packages. This limited transparency and accessibility can complicate scientific advances in the fields of GC × GC and chemometrics. In response, some research groups are developing open‐access alternatives. These have mostly been reviewed recently by Bos et al. [151] and Stefanuto et al. [143]. One recent and interesting development is the DA_2Dchrom package by Ladislavová et al. [164], who packed many of the recent spectral‐data‐alignment tools in an open‐source toolbox that is available online. The toolbox includes their own TNT‐DA algorithm [164]. Similar to Niezen et al., the authors pointed out that different algorithms perform better under different conditions, and their tool is meant to aid researchers in quickly assessing the different tools and evaluating their performance using their own data.

After alignment, normalization and scaling (or transformation) of the data can be conducted. A recent review of this step is still up‐to‐date [151].

### 5.2 Nontargeted analysis

Most recent developments focus on nontargeted analysis. Contrary to targeted analysis, nontargeted analysis (sometimes also referred to as untargeted analysis) does not rely on a list of compounds of interest. Therefore, it requires an elaborate approach.

To simplify the data problem, untargeted approaches typically employ dimension‐reduction algorithms [165]. There are a number of ways to feed the data to the untargeted‐analysis algorithms, generally categorized into three main strategies: pixel‐based, peak‐table, and tile‐based approaches.

The simplest of these three is the pixel‐based approach, which, as implied, divides the 2D chromatogram into pixels. Each pixel is then treated as a single variable. The major advantage of this approach is that raw data can be directly processed as collected by the instrument [142]. As earlier mentioned, scaling prior to pixel‐based analysis is essential to remove nonsample variation and improve the performance and reliability of the analysis [166]. However, this approach is quite susceptible to false positives, commonly caused by detector fluctuations and misalignments. This is because large fluctuations can be hard to distinguish from small peaks [167, 168]. Additionally, pixel‐based data analysis is computationally expensive, making it harder to use when dealing with large datasets [142]. To manage large datasets, an alternative strategy involves reducing the large data sets to peak tables. For table‐based methods, alignment of the peaks is a very important step in preprocessing to prevent multiple entries for a single peak [142, 169]. A disadvantage of this approach is the time‐consuming process of identifying peaks prior to constructing peak tables.

As a compromise between the peak‐table and the pixel‐based methods, the group of Synovec developed the tile‐based Fisher‐ratio approach [142]. Here, the 2D data is binned using a tile‐based approach. This eliminates the need for 2D alignment. The method yields a greater probability of true positives than of false positives. The chromatogram is divided in such a way that a tile encompasses an entire peak in both dimensions. Tile‐based analysis is advantageous, because it is not vulnerable to misalignment and inherently involves data reduction. In a recent work, Trinklein and Synovec studied the effect of chromatographic saturation, retention time shifts, and within‐class signal variance on the optimal tile size [168].

## 6 GENERAL DISCUSSION AND CONCLUSIONS

GC × GC has developed dramatically in the three decades since the technique was first demonstrated experimentally. The number of users, published methods, and scientific papers has increased dramatically.

Quite an exciting picture arises when we consider the progress made on GC × GC modulation techniques in the last 5 or 10 years. The following conclusions can be drawn (with the most important one listed first.

1. Quite significant progress has been and is being made.
2. Thermal modulators yield excellent performance, even without the need for coolant consumables.
3. Novel flow modulators can compete with thermal modulators, yet tend to be simpler and easier to operate.

An inventory of recent papers indicates that overall thermal modulation is used in two‐thirds of all cases. However, of the application‐oriented papers some 90% employ thermal modulation, while more than 60% of technology‐focused papers involve flow modulation. If recent research is an indication of future applications, we may see a shift towards flow modulation in the years to come. This may be aided by tools that facilitate a transfer of methods from thermal modulation to flow modulation systems [170]. The discrepancy between research and application underlines that GC × GC is an important and established technique that is still immature and likely to improve further in the years to come.

Detection for GC × GC appears to be making a major swing towards MS. This is certainly true in research environments, as is evident from the scientific literature. However, GC × GC is especially useful for (untargeted) fingerprinting. For targeted analysis of limited numbers of analytes, it remains to be seen whether—or how often—GC × GC can compete with GC‐MS. For fingerprinting (semi‐) universal detectors, such as the FID, or element‐specific detectors, such as the SCD, remain attractive.

Developing and optimizing GC × GC methods is a complex task, for which currently few tools are available. Software that aids in selecting column dimensions and flow and/or pressure settings is very helpful in this context. Analyte‐dependent optimization of separations is even more challenging. Currently, retention‐modeling software tools are still immature and unavailable to the general (analytical) public. Much research is still needed to fill a blatant need.

Data analysis continues to be an important aspect of GC × GC. There is still a large ongoing scientific effort, but it is difficult to evaluate the various contributions. This would require benchmark data sets. There is a trend to realize this through realistic simulated data.

## CONFLICT OF INTEREST

The authors declare no conflict of interest.

## Data Availability

Data sharing is not applicable to this article as no new data were created or analyzed in this study.
